# Epidemiology of patients presenting to a pediatric emergency department in Karachi, Pakistan

**DOI:** 10.1186/s12873-018-0175-4

**Published:** 2018-08-03

**Authors:** Nadir Ijaz, Matthew Strehlow, N. Ewen Wang, Elizabeth Pirrotta, Areeba Tariq, Naseeruddin Mahmood, Swaminatha Mahadevan

**Affiliations:** 1 Department of Emergency Medicine, Stanford University School of Medicine, 300 Pasteur Dr, Rm M121, Alway Building MC 5119, Stanford, CA 94305 USA; 2 Honors Program in Medical Education, Northwestern University, Evanston, IL USA; 3 Department of Paediatrics and Child Health, Aga Khan University, Karachi, Pakistan

**Keywords:** Global health, Pediatric emergency medicine, Epidemiology, Outcomes, Pakistan

## Abstract

**Background:**

There is little data describing pediatric emergencies in resource-poor countries, such as Pakistan. We studied the demographics, management, and outcomes of patients presenting to the highest-volume, public, pediatric emergency department (ED) in Karachi, Pakistan.

**Methods:**

In this prospective, observational study, we approached all patients presenting to the 50-bed ED during 28 12-h study periods over four consecutive weeks (July 2013). Participants’ chief complaints and medical care were documented. Patients were followed-up at 48-h and 14-days via telephone.

**Results:**

Of 3115 participants, 1846 were triaged to the outpatient department and 1269 to the ED. Patients triaged to the ED had a median age of 2.0 years (IQR 0.5–4.0); 30% were neonates (< 28 days). Top chief complaints were fever (45.5%), diarrhea/vomiting (32.3%), respiratory (23.1%), abdominal (7.5%), and otolaryngological problems (5.8%). Temperature, pulse and respiratory rate, and blood glucose were documented for 66, 42, and 1.5% of patients, respectively. Interventions included medications (92%), IV fluids (83%), oxygen (35%), and advanced airway management (5%). Forty-five percent of patients were admitted; 11 % left against medical advice. Outcome data was available at time of ED disposition, 48-h, and 14 days for 83, 62, and 54% of patients, respectively. Of participants followed-up, 4.3% died in the ED, 11.5% within 48 h, and 19.6% within 14 days.

**Conclusions:**

This first epidemiological study at Pakistan’s largest pediatric ED reveals dramatically high mortality, particularly among neonates. Future research in developing countries should focus on characterizing reasons for high mortality through pre-ED arrival tracking, ED care quality assessment, and post-ED follow-up.

**Electronic supplementary material:**

The online version of this article (10.1186/s12873-018-0175-4) contains supplementary material, which is available to authorized users.

## Background

Pakistan is a resource-poor country in South Asia that has recently taken significant steps to improve its national healthcare delivery. Despite its efforts, Pakistan continues to perform poorly on many international health indicators, especially with regard to its pediatric population. In 2016, its neonatal, infant, and under-five mortality rates were 46, 64, and 79 per 1000 live births, respectively, giving Pakistan the world’s highest neonatal mortality rate, as well as the 11th highest infant and 20th highest under-five mortality rates [1]. One contributor to high pediatric morbidity and mortality in Pakistan is the absence of an organized emergency care system, which could account for nearly half of deaths and one-third of disability-adjusted life years (DALYs) [2].

Recent work in Pakistan has identified serious issues which hinder access to quality emergency care, including overall dissatisfaction with emergency medical services, low staff confidence in their ability to handle emergencies, and a general shortage of essential equipment and supplies [3]. While improving access to quality emergency care is recognized internationally as an important step in reducing morbidity and mortality in low- and middle-income countries (LMICs) [4], there is a dearth of data describing the epidemiology of patients seeking emergency care in Pakistan, particularly pediatric patients.

Characterization of patients seeking emergency care services is essential to determine a country’s emergency care resource needs and develop a locally appropriate emergency healthcare system [5]. The 2013 Academic Emergency Medicine consensus conference, “Global Health and Emergency Care: A Research Agenda,” highlighted the lack of published data on chief complaints as a critical gap in global emergency care research [6]. We seek to bridge this gap by describing the epidemiology and outcomes of children presenting to the emergency department of Pakistan’s largest government children’s hospital, the National Institute of Child Health (NICH).

## Methods

### Study design and setting

The study was conducted in the emergency department (ED) of a public, tertiary care, pediatric hospital in Karachi, Pakistan. The 500-bed hospital houses a 50-bed ED run as a public-private partnership between the hospital and the ChildLife Foundation (CLF), a local nonprofit organization focused on quality improvement in pediatric emergency care. The facility mainly serves the surrounding urban population, with a small percentage of patients coming from rural areas in the southern provinces of Sindh and Balochistan. While there are other private hospitals in the area, it remains the sole government healthcare facility that specializes in the delivery of pediatric care in Karachi, a city of 14.9 million people [7].

### Selection of participants

All patients presenting to the ED waiting room for an unscheduled visit were approached for enrollment during 28 12-h study periods spanning four consecutive weeks: two weeks of daytime (8 AM-8 PM) enrollment followed by two weeks of nighttime (8 PM-8 AM) enrollment from July 1–28, 2013. Upwards of 90% of patients consented to participate. Patients with parents/guardians who did not speak Urdu (< 3%) were excluded due to lack of translation services.

### Data collection and measurements

After obtaining verbal consent, trained research assistants collected demographic and clinical data using a standardized, secure, web-based survey form in Research Electronic Data Capture (REDCap). Paper questionnaires served as backups when necessary, and any data recorded on paper was later entered into REDCap using a double data entry procedure.

An ED nurse obtained chief complaint information and vital signs in the waiting room prior to triaging patients either to the outpatient department (OPD, a separate section of the ED open 24 h per day for urgent care) or to ED treatment areas. Patient gender, age, and up to three chief complaints were recorded for all patients. If a patient was triaged to the OPD, no further information was collected and initial vital signs were not recorded.

Clinical information was obtained from ED medical records and, for admitted patients, from inpatient records. Medical records were paper-based. Vital signs were documented as “unmeasured” if not recorded by the triage nurse or during initial ED evaluation. In situations where multiple modes of transportation were needed to reach the hospital, only the final mode of transportation was recorded. Patients were classified as underweight if their weight fell below the 3rd percentile using World Health Organization (WHO) weight-for-age standards.

Patients were followed-up by examination of discharge or admission records and by telephone at 24–72 h and 14 days following their initial ED visit. If medical records were not locatable at the end of their ED course, patients were deemed lost to follow up at time of ED disposition. Patients unable to provide contact phone numbers, those who provided invalid phone numbers, and those who were contacted twice without response were deemed lost to follow-up. Some patients unable to be reached at 24–72 h were successfully contacted at 14 days. Limited disposition information was gathered at 48-h and 14-day phone follow-up for patients for whom discharge records were unavailable, and this information was used to determine their ED disposition status.

### Outcomes

Measured outcomes included mortality, hospital admission, and guardian-reported functional status relative to baseline at 48-h and 14-days after initial presentation to the hospital.

### Primary data analysis

Categorical demographic and clinical characteristics were assessed with frequency tabulations and chi-squared tests; for age groups and sex, odds ratios were calculated through descriptive univariate logistic regression models. Summary statistics were calculated for continuous variables, and are presented as sample medians and interquartile ranges (IQR). Two descriptive multivariate logistic regression models were constructed with primary outcomes of admission and mortality using independent variables identified as significant with *p*-values of less than or equal to 0.2 through univariate analysis. Predictors in the multivariate logistic regression models with p-values of less than or equal to 0.05 was deemed significant. Models controlled for gender and date of ED arrival, and model fit was evaluated with the c-statistic. The admission model of patients ages >than 28 days used 87% of available records, c = 0.77. The 14-day mortality model used 85% of available records, c = 0.82. SAS Enterprise Guide software, Version 4.3, was used for statistical analysis.

## Results

### Comparison of OPD and ED patients

**Fig. 1:**
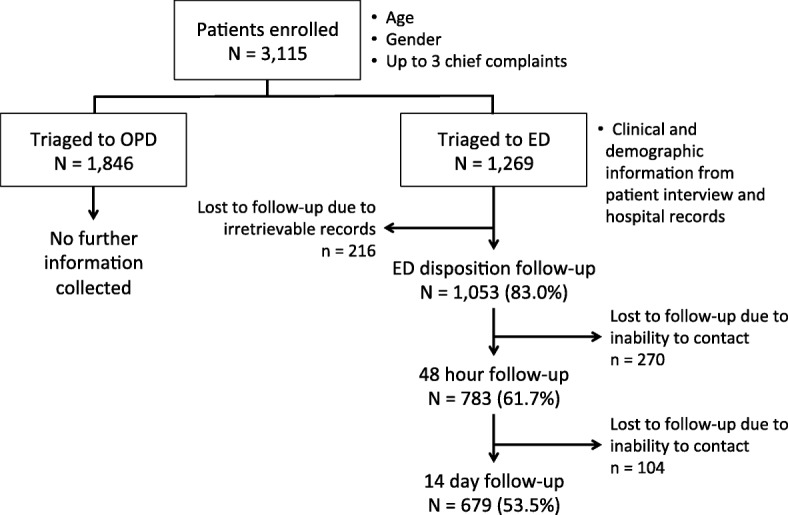
Study design and follow-up rates

During the 28-day study period, 3115 patients were enrolled. Forty-one percent (*n* = 1269) of these patients were triaged to the ED; the remaining 1846 patients were directed to the OPD (Fig. 1). Enrolled patients ranged in age from 0 to 13 years, with a median age of 2.0 years (IQR 0.5–4.0). ED patients were significantly younger than OPD patients, with neonates and infants more likely to be triaged to the ED in comparison to children older than 5 years (Table 1).

**Table 1:** Demographics of OPD and ED visits

	All visits, *N* (%)	ED visits, *N* (%)	OPD visits, *N* (%)	Odds ratio^a^
No. of visits	3115	1269 (40.7)	1846 (59.3)	–
Age, yrs., median (IQR)	2.0 (0.50–4.0)	0.83 (0.03–3.0)	2.0 (0.92–5.0)	0.90 (0.88–0.93)
Neonate (<28d)	439 (14.1)	378 (29.8)	61 (3.3)	12.7 (9.3–17.4)
Infant (28d- < 1 yr)	672 (21.6)	271 (21.4)	401 (21.7)	1.4 (1.1–1.7)
Young child (1-3 yrs)	778 (25.0)	245 (19.3)	533 (28.9)	0.9 (0.8–1.2)
Child (3-5 yrs)	470 (15.1)	123 (9.7)	347 (18.8)	0.7 (0.6–0.9)
Older child (> 5 yrs)	746 (24.0)	244 (19.3)	502 (27.2)	Reference
Sex				
Female	1303 (41.8)	514 (40.5)	789 (42.7)	Reference
Male	1808 (58.0)	754 (59.4)	1054 (57.1)	1.1 (0.9–1.3)

^a^Odds ratios compare likelihood of patient triage to ED instead of OPD using indicated reference groups

### ED patient presentation

Only 22.0% of patients arrived at the hospital by ambulance (including both pre-hospital care and transfers from other healthcare facilities). Median patient travel time was 30 min (Table 2). Almost two-thirds of patients (64.2%) had sought medical care for their current condition prior to their arrival in the ED, with 7.3% being transferred from another health care facility, predominantly by ambulance. An additional 38.9% of patients were referred or advised to come to the hospital by an outside provider (Table 2).

**Table 2:** ED patient characteristics

Characteristic	No. ED visits (%)^a^ (*N* = 1269)
Travel to hospital	
Mode of transport	
Taxi/rickshaw	421 (33.2)
Ambulance	279 (22.0)
Motorbike	190 (15.0)
Bus	127 (10.0)
Other^b^	114 (9.0)
Travel time, median (IQR), min	30 (20–60)
Illness timeline	
Symptom duration (before ED visit)	
Sudden (< 24 h)	481 (37.9)
Recent (1–3 days)	340 (26.8)
Subacute (4–14 days)	245 (19.3)
Chronic (> 2 weeks)	96 (7.6)
Time before seeking any medical care^c^	
< 24 h	381 (30.0)
≥ 24 h	274 (21.6)
Time of ED presentation (HH:MM)	
00:00–05:59	193 (15.2)
06:00–11:59	339 (26.7)
12:00–17:59	442 (34.8)
18:00–23:59	295 (23.3)
Prior Care	
No prior care	331 (26.1)
Referral from another provider	494 (38.9)
Returning to same hospital	123 (9.7)
Direct transfer from another provider	92 (7.3)
Vital signs^d^	
Prior care without referral	105 (8.3)
Abnormal (≤36 °C or ≥ 38 °C)	105 (8.3)
Unmeasured	430 (33.9)
Pulse	
Abnormal^e^	153 (12.1)
Unmeasured	727 (57.3)
Respiratory rate	
Abnormal^e^	327 (25.8)
Unmeasured	736 (58.0)
Blood pressure	
Abnormal^f^	Not calculated
Unmeasured	1259 (99.2)
Pain present	380 (30.0)
Prior health status	
Underweight^g^	421 (33.2)
Neonates, *n* = 378	
Preterm^h^	85 (22.5)
Low birth weight^i^	97 (25.7)
Age ≥ 28 days, *n* = 883	
No vaccinations	91 (10.3)

^a^Not all categories sum to 100% as some values are missing

^b^Other modes of transport included private car, bicycle, and walking

^c^Time lapse between onset of illness/injury episode and visit to first healthcare provider

^d^“Unmeasured” vitals were either not measured or not recorded by ED staff

^e^Abnormal pulse and respiratory rate were classified based on patient age

^f^Blood pressure was measured in too few patients (*n* = 10) to draw clinical conclusions

^g^Defined as <3rd percentile on WHO weight-for-age curves

^h^Defined as > 37 weeks gestational age, as reported by parent/guardian

^i^Defined as weight < 2500 g at birth, as reported by parent/guardian

Documentation of patient vital signs by medical staff was inconsistent. Temperature was recorded in two-thirds of visits, with all other vital signs charted in less than half of patients. Blood pressure was seldom charted (0.8%). Respiratory rate was the most frequent abnormal vital sign, with a quarter of patients presenting with an abnormal value (Table 2).

### ED patient chief complaints

**Fig. 2:**
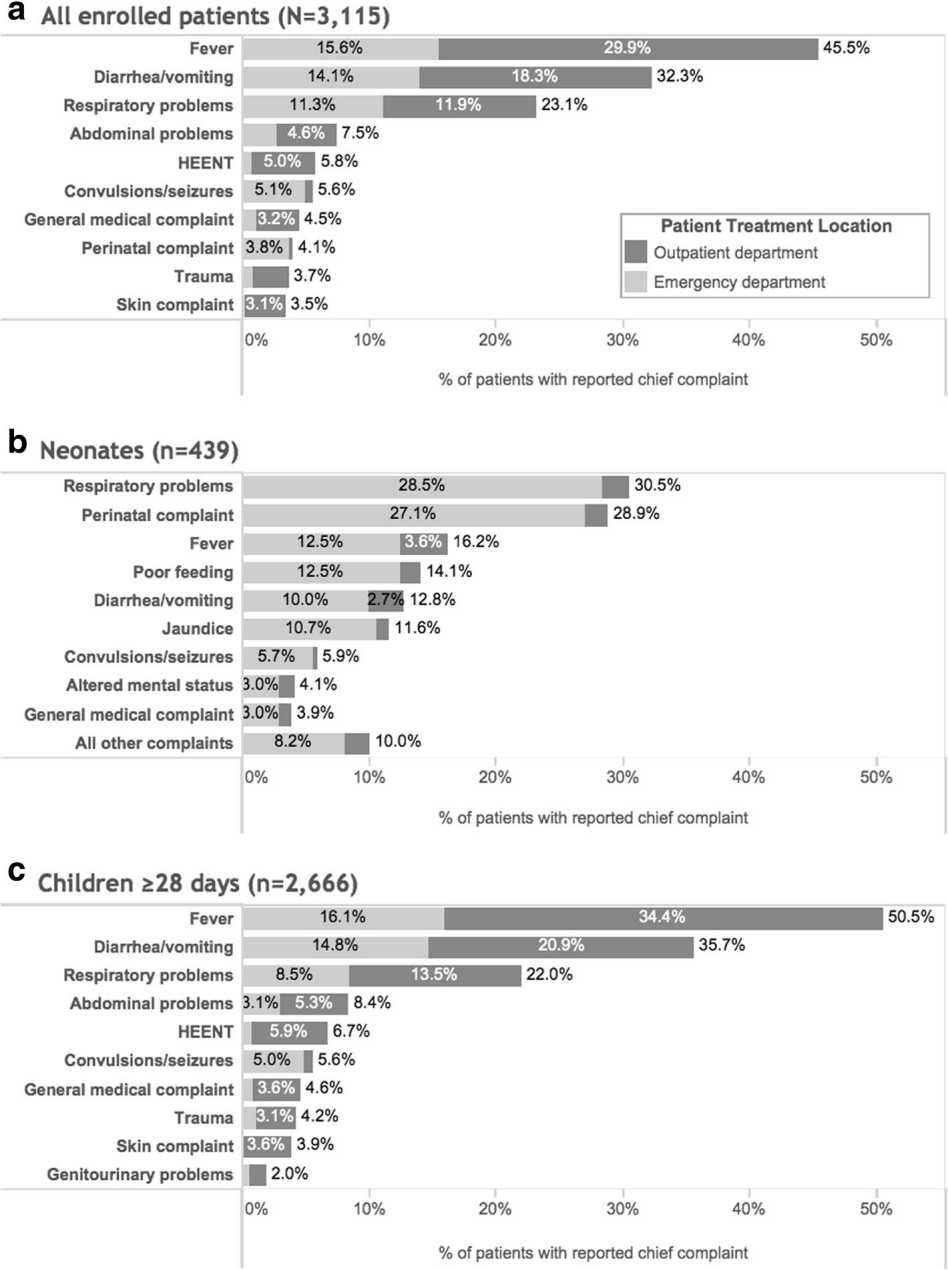
Common chief complaints by location of treatment for (**a**) all enrolled patients (*N* = 3115), (**b**) neonates (*n* = 439), and (**c**) patients ≥28 days (*n* = 2666)

For all enrolled ED pediatric patients, the most common chief complaints (not mutually exclusive) were fever (45.5%), diarrhea/vomiting (32.3%), and respiratory problems (23.1%). For neonates, top chief complaints were respiratory problems (30.5%), perinatal complaints defined as complications at birth or referral by birth attendant (28.9%), and fever (16.2%). For patients ≥28 days, the top three complaints aligned with those for all patients: fever (50.5%), diarrhea/vomiting (35.7%), and respiratory problems (22.0%) (Fig. 2).

### ED therapeutic or procedural interventions

Nearly all patients (92.2%) received medications during their visit. Antibiotics, nebulized medications, and antipyretics (including acetaminophen and non-steroidal anti-inflammatory drugs) were the most common medication types, prescribed to 43.3, 10.9, and 10.7% of patients, respectively. All other medication types (e.g. other analgesic, antimalarial) were prescribed to less than 10% of patients. The second most common intervention was intravenous (IV) access with IV hydration, which was utilized in 82.7% of patients. More than one-third of patients (35.4%) received some form of supplemental oxygen, with 4.6% of patients receiving an advanced airway intervention. Nasogastric tubes were placed in 10.3% of patients.

### ED patient disposition and outcomes

Of the 1269 patients triaged to the ED, 1053 (83.0%) patients were followed-up at the time of ED disposition (Table 3). Of those patients followed up, 44.7% were admitted to either the hospital ward or ICU, 24.7% were discharged to home, 10.9% left against medical advice, and 13.9% did not have a recorded reason for leaving. Of the 349 patients admitted from the ED whose hospital location was recorded, 16.3% had an ED arrival to disposition time of at least 6 h and 21.1% spent the night in the ED, defined as arriving before 12 AM and time of disposition after 7 AM. We created a multivariate model to identify predictors of admission; significance was only achieved with children older than 1 year. In this sub-analysis admission model, abnormal level of consciousness by AVPU, respiratory complaint, prior care, and care during Ramadan were associated with higher likelihood of admission, while age younger than 6 was associated with lower likelihood of admission (Additional file 1: Table S1).

**Table 3:** Patient disposition and outcomes at 48 h and 14 days with comparison of neonates and children ≥28 days

Disposition and Outcomes^a^	All ED visits	Neonatal visits	Children ≥ 28 days	Odds ratio (comparing neonates vs. children ≥ 28 days)
	*N* (%)	*N* (%)	*N* (%)	
No. of total ED visits	1269	378	883	
ED Disposition				
Followed up at time of ED disposition^b^	1053 (83.0)	333 (88.1)	714 (80.9)	–
Admitted	471 (44.7)	194 (58.3)	272 (38.1)	3.5 (2.4–5.1)
Home	528 (50.1)	111 (33.3)	416 (58.3)	
Left against medical advice	115 (10.9)	51 (15.3)	64 (9.0)	3.9 (2.4–6.4)
Left before seen by provider	7 (0.7)	3 (0.9)	4 (0.6)	3.7 (0.8–17.0)
Discharged home by provider	260 (24.7)	44 (13.2)	215 (51.7)	Reference
Unknown reason for leaving	146 (13.9)	13 (3.9)	133 (18.6)	0.5 (0.2–0.9)
Transferred out	4 (0.4)	1 (0.4)	3 (0.4)	1.6 (0.2–16.0)
Died in ED	45 (4.3)	26 (7.8)	19 (2.7)	6.7 (3.4–13.1)
48-h follow-up				
Followed up at 48 hours^b^	783 (61.7)	252 (66.7)	526 (59.6)	–
Returned to normal function^c^	277 (35.4)	70 (27.8)	206 (39.2)	0.8 (0.5–1.1)
Still in original hospital	197 (25.2)	76 (30.2)	119 (22.6)	2.4 (1.6–3.5)
Returned to care^d^	167 (21.3)	43 (17.1)	123 (23.4)	1.1 (0.7–1.8)
Surgery by 48 h	20 (2.6)	5 (2.0)	15 (2.9)	0.8 (0.3–2.2)
Cumulative deaths by 48 h	90 (11.5)	48 (19.0)	41 (7.8)	2.8 (1.8–4.4)
14-day follow-up				
Followed up at 14 days^b^	679 (53.5)	225 (59.5)	450 (51.0)	–
Returned to normal function^c^	420 (61.9)	115 (51.1)	303 (67.3)	1.0 (0.6–1.5)
Still in original hospital	37 (5.4)	11 (4.9)	25 (5.6)	1.2 (0.6–2.4)
Returned to care^d^	257 (37.8)	80 (35.6)	177 (39.3)	1.1 (0.8–1.6)
Surgeries, 48 hours - 14 days	18 (2.7)	4 (1.8)	14 (3.1)	0.6 (0.2–2.2)
Cumulative deaths by 14 days	133 (19.6)	75 (33.3)	57 (12.7)	3.4 (2.3–5.1)

^a^Not all categories sum to 100% as some values are missing

^b^For these rows, percentages are out of the number of total ED visits

^c^“Return to normal function” is guardian’s perception of recovery relative to child’s baseline

^d^“Return to care” is guardian’s report of returning to medical care after leaving hospital

Follow-up rates were 61.7% at 48 h and 53.5% at 14 days; 19.0% of ED patients lacked telephone access. Almost two-thirds of followed patients reported a return to normal functional level at 14 days (Table 3). Forty-five patients died while in the ED, comprising 4.3% of the 1053 patients followed-up at the time of ED disposition (Table 3). At the 48-h follow-up, 45 more patients had died for an overall 48-h mortality rate of 11.5%. An additional 43 patients died by 14 days for a cumulative mortality rate of 19.6%. Of patients who left against medical advice, 12.2% died within 14 days. Of patients who were discharged or left the ED and subsequently died, 60% returned to care before death. Of deaths that occurred within 48 h after ED disposition, 13% occurred at home; within 14 days, 31% occurred at home. Neonates were more likely to be admitted, leave against medical advice, and die as compared to their older counterparts. A multivariate model for predictors of cumulative mortality at 14 days identified neonate status, abnormal AVPU, respiratory complaint, underweight for age, ambulance arrival, and prior care as being associated with higher likelihood of mortality; fever complaint and care during Ramadan were protective (Additional file 2: Table S2).

## Discussion

This report is the first in-depth investigation of the epidemiology of patients presenting to a pediatric emergency department in Pakistan and one of only a handful of such studies conducted to-date in developing countries [8–11]. Prior studies in Pakistan have focused primarily on the epidemiology of pediatric traumatic injuries, which account for only 3.7% of patients in our study [12]. We sought to characterize the population of all pediatric patients seeking acute care in Pakistan and found a high burden of infant visits and an alarming cumulative 14-day mortality rate, which was particularly high among neonates.

In comparison to urban pediatric emergency departments in other LMICs, our overall reported pediatric mortality rate (19.6%) was exceedingly high, with a full one-third (33.3%) of neonates having died within 14-days of their ED visit. In comparison, an Indian study reported a substantially lower in-hospital mortality rate of 12.2%, although this number does not include patients who were sent home from the ED. [13] In our study, 4.3% of children died while in the ED, a similar rate to that published in a separate study (5.7%) of Indian children between 1995 and 2000 [8]. Dramatically lower ED mortality rates have been reported in Egypt (0.9%) and Turkey (2.6%), an unexpected contrast given their similar resource and training limitations [9, 14]. Pakistan’s high death rates also stand in sharp contrast to the numbers reported from countries with developed emergency care infrastructures. Studies in the United States demonstrate few deaths in the ED, with a < 0.1% mortality rate among pediatric patients [15]. To our knowledge, there are no other studies that track all patients following ED discharge to assess for delayed mortality. As the majority of deaths in our study occurred after the patient left the ED (a post-ED mortality rate of 15.3%, accounting for 66% of deaths), our study highlights a need to monitor the progression of patients following ED disposition.

Many factors likely contribute to the exceedingly high mortality rate we observed. First and foremost, delays in care seeking likely exacerbated delays in reaching the appropriate healthcare facility. One study of Pakistani households reported that less than half of family members with emergency medical conditions were taken to the nearest primary care facility for treatment [16]. In our study, almost two-thirds of our patients were seen by outside health care providers prior to their arrival in the ED, but only a quarter (22.0%) were transferred by ambulance from that initial medical facility. Most patients travelled to the ED via public or private vehicle, therefore benefiting from neither the speed nor the en route emergency medical treatment that ambulance transport provides. Our results, therefore, complement the findings of a prior study that described prolonged travel times to the appropriate health care facility as an important contributor to pediatric deaths in Karachi [17].

Most of our study deaths occurred following ED evaluation and disposition, suggesting the failure of ED medical staff to recognize illness severity and promptly initiate life-saving therapy [18]. One study in northern Pakistan found that misinterpretation of symptom severity by the initial providers and mis-triage at the initial medical facility led to poor outcomes in many cases of surgically treatable illnesses [19]. Despite vital signs being important markers of illness acuity and reliable predictors of death in emergency settings [20], initial vital signs were not reliably documented or reported at our study hospital. Obtaining a complete set of vital signs and implementing effective ED triage have been shown to reduce mortality in resource-poor settings [21]. Our results suggest that improved training for Pakistani medical staff on the importance of taking and documenting vital signs may lead to the proper identification of the most severely ill patients, thus reducing overall mortality.

The observed elevated mortality rates are also likely impacted by both a lack of essential equipment and inadequate physician education regarding its use when available [22, 23]. Research has identified significant gaps in the availability of essential emergency equipment and medications, as well as the knowledge necessary for Pakistani physicians to use these resources effectively in relevant settings [22]. Remarkably, much of the frequently unavailable equipment, such as suction catheters and pediatric-sized blood pressure cuffs, consists of relatively inexpensive items capable of saving lives in emergency scenarios.

Finally, a significant percentage (10.9%) of patients left against medical advice (AMA). As one-eighth (12.2%) of these children died within 14 days of their initial presentation, clearly leaving AMA was a significant contributor to our high mortality rates. This behavior has previously been identified as a common problem in Karachi hospitals [24]. Many parents and guardians cited delays in care and inadequate communication from hospital staff about the ED process as reasons for leaving the hospital prematurely. It has been shown that leaving AMA is associated with higher mortality and readmission rates [25]. Therefore, improving patient-provider communication and the hospital efficiency should be further studied as these factors may be contributing to families leaving AMA.

The most common chief complaints (fever, diarrhea/vomiting, and respiratory problems) reported during our study were similar, but varied in frequency, to those previously reported in other developing countries [8–11]. Respiratory (23.4%) and gastrointestinal illness (23.3%) accounted for almost half of visits to a pediatric ED in India [8]. In Egypt and Tunisia, respiratory complaints accounted for 41.7 and 39.9% of visits, respectively [9, 10]. Respiratory complaints may have been lower in our study because our data was collected during the summer months, when such illness tend to diminish. While injuries were the most common complaint for pediatric ED patients in a Malaysian study, fever (24%), breathing difficulties (16.6%), and diarrhea/vomiting (9.7%) were the next most frequent presentations [11]. The observed trend of similar chief complaints among LMICs in connection with high mortality rates suggests that focusing on these specific chief complaints in developing provider training materials and patient management protocols may be effective in reducing mortality. A recent WHO study also reports that the majority ED providers in LMICs have no specialty training in emergency medicine [26]. Additional study exploring the training local providers receive on managing chief complaints associated with high mortality would be of further benefit.

Our study documents a staggeringly high mortality rate at Pakistan’s largest children’s emergency department. Addressing this mortality rate, which even significantly exceeds other comparable resource-limited countries, is crucial for Pakistan to achieve its goal of improving its national healthcare delivery. Focusing training on the most common regional chief complaints may ultimately improve patient management and outcomes. Additional studies are needed to further characterize which factors – delays in seeking and accessing care, inadequate triage, gaps in physician education, lack of necessary equipment, or others – are the greatest contributors to the high mortality observed at our study site. Likewise, investigating the quality of care patients received and the appropriateness of discharge in patients who were not admitted is vital to decreasing morbidity and mortality at the National Institute of Child Health in Karachi and other similar hospitals.

### Limitations

While this is the first, large-scale pediatric emergency medicine epidemiological study in Pakistan, we had relatively low 48-h and 14-day follow-up rates, largely due to poor medical record-keeping and patients’ lack of phone access. Secondly, three out of four weeks of data collection occurred during the religious month of Ramadan. As a result, follow-up data collection was challenging during the hours (8–9:30 PM) when fasting had concluded for the day. Additionally, it is possible that we captured some trends unique to the month of Ramadan that are different from the remainder of the year. It is unclear what effect Ramadan would have on our data.

## Conclusions

This first investigation of pediatric emergency epidemiology in Pakistan has provided a broader understanding of the most common presentations and resulting outcomes. Our study’s findings can be used to inform quality improvement and policy changes in ED management, create guidelines and needs-based training materials for local healthcare providers, and serve as a baseline for measurement of future trends in health outcomes. Future work should focus on identifying high-risk patients earlier in their clinical course by improving triage, training staff to increase their competency in managing the most common chief complaints, especially neonatal complaints, improving local prenatal and neonatal care, and assessing the impact of such interventions on morbidity and mortality. Much of the knowledge gained from this study will have applicability and value to other developing nations facing similar challenges.

## Additional files

Additional file 1: **Table S1.** Logistic regression model for predictors of admission for children greater than 1 year of age (*N* = 470, c-statistic = 0.77): 85% of followed patients were included in the model. Of these patients, 36% were admitted, 64% were sent home. Model was controlled for gender. (DOCX 17 kb)

Additional file 2: **Table S2.** Logistic regression model for predictors of mortality for all patients (*N* = 679, c-statistic = 0.82): 85% of followed patients were included in this model, of which 17% of patients died. Model was controlled for gender. (DOCX 15 kb)

## Abbreviations

AMA: Against medical advice
CLF: ChildLife Foundation, Pakistan
DALY: Disability-adjusted life year
ED: Emergency department
HEENT: Head, eyes, ears, nose, and throat
IV: Intravenous
LMICs: Low and middle-income countries
NICH: National Institute of Child Health, Karachi, Pakistan
OPD: Outpatient department
REDCap: Research Electronic Data Capture
WHO: World Health Organization
