# Correction to: Epidemiology of patients presenting to a pediatric emergency department in Karachi, Pakistan

**DOI:** 10.1186/s12873-020-00364-5

**Published:** 2020-08-28

**Authors:** Nadir Ijaz, Matthew Strehlow, N. Ewen Wang, Elizabeth Pirrotta, Areeba Tariq, Naseeruddin Mahmood, Swaminatha Mahadevan

**Affiliations:** 1 Department of Emergency Medicine, Stanford University School of Medicine, 300 Pasteur Dr, Rm M121, Alway Building MC 5119, Stanford, CA 94305 USA; 2 Honors Program in Medical Education, Northwestern University, Evanston, IL USA; 3 Department of Paediatrics and Child Health, Aga Khan University, Karachi, Pakistan

**Correction to: BMC Emerg Med 18, 22 (2018)**

**https://doi.org/10.1186/s12873-018-0175-4**

The ethics statement in this article [1] incorrectly states that the study was approved by the IRB of the National Institute of Child Health (NICH) in Karachi. Contrary to this statement, the authors received approval to complete the study from the director of the NICH, not the ethics committee. The authors also received approval from the IRB of Stanford University School of Medicine, who have confirmed that their ethics approval remains valid without approval from the IRB at the NICH in Karachi. The IRB at the NICH in Karachi has confirmed that the authors should have applied for ethics approval for this study and that they cannot provide retrospective approval. Given the non-invasive nature of the study, the opinion of the Stanford University School of Medicine IRB and the potential public health implications of the findings, the Editor and Publisher have decided to take no further action.

## References

[1] Ijaz N, Strehlow M, Wang NE, Pirrotta E, Tariq A, Mahmood N, Mahadevan S (2018). Epidemiology of patients presenting to a pediatric emergency department in Karachi, Pakistan. BMC Emerg Med.

