# Supplementary material for: Epidemiology of patients presenting to a pediatric emergency department in Karachi, Pakistan
Source: BMC Emerg Med. 2018 Aug 3;18:22. doi: 10.1186/s12873-018-0175-4 (PMC6091113; doi:10.1186/s12873-018-0175-4)
Supplement: Supplementary file 1 — Table S1. Logistic regression model for predictors of admission for children greater than 1 year of age (N = 470, c-statistic = 0.77): 85% of followed patients were included in the model. Of these patients, 36% were admitted, 64% were sent home. Model was controlled for gender. (DOCX 17 kb) [file 12873_2018_175_MOESM1_ESM.docx]

Additional file 1: Table S1. Logistic regression model for predictors of admission for children greater than 1 year of age (N = 470, c-statistic = 0.77): 85% of followed patients were included in the model. Of these patients, 36% were admitted, 64% were sent home. Model was controlled for gender.

| **Predictors** | **Odds Ratio** | **95% Confidence Limits** |
| --- | --- | --- |
| Age |  |  |
| **Young child (1-3 yrs)** | **0.39** | **[0.19, 0.78]** |
| **Child (3-5 yrs)** | **0.50** | **[0.27, 0.94]** |
| Older Child (6-13 yrs) | reference |  |
| Arrival information |  |  |
| **Arrived during Ramadan** | **2.88** | **[1.18, 7.00]** |
| Transport time to hospital, min | 1.01 | [1.00, 1.01] |
| Transported by ambulance | 1.55 | [0.85, 2.82] |
| **Prior care sought** | **2.39** | **[1.39, 4.14]** |
| Clinical Presentation |  |  |
| Fever | 1.28 | [0.80, 2.04] |
| Vomiting/diarrhea complaint | 0.60 | [0.34, 1.04] |
| **Respiratory complaint** | **3.13** | **[1.65, 5.92]** |
| Seizure complaint | 0.95 | [0.49, 1.83] |
| **Abnormal AVPU** | **2.97** | **[1.70, 5.20]** |
| Underweight | 1.22 | [0.57, 2.64] |
