# Supplementary material for: Epidemiology of patients presenting to a pediatric emergency department in Karachi, Pakistan
Source: BMC Emerg Med. 2018 Aug 3;18:22. doi: 10.1186/s12873-018-0175-4 (PMC6091113; doi:10.1186/s12873-018-0175-4)
Supplement: Supplementary file 2 — Table S2. Logistic regression model for predictors of mortality for all patients (N = 679, c-statistic = 0.82): 85% of followed patients were included in this model, of which 17% of patients died. Model was controlled for gender. (DOCX 15 kb) [file 12873_2018_175_MOESM2_ESM.docx]

Additional file 2: Table S2. Logistic regression model for predictors of mortality for all patients (N = 679, c-statistic = 0.82): 85% of followed patients were included in this model, of which 17% of patients died. Model was controlled for gender.

| **Predictors** | **Odds Ratio** | **95% Confidence Limits** |
| --- | --- | --- |
| **Neonate < 28 days old** | **1.93** | **[1.10, 3.39]** |
| Arrival information |  |  |
| **Arrived during Ramadan** | **0.33** | **[0.12, 0.93]** |
| Transport time to hospital, min | 1.00 | [0.99, 1.00] |
| Transported by ambulance | **3.02** | **[1.80, 5.07]** |
| **Prior care sought** | **1.96** | **[1.03, 3.75]** |
| Clinical Presentation |  |  |
| Symptom onset < 24 hrs | 1.20 | [0.67, 2.12] |
| **Fever** | **0.51** | **[0.27, 0.95]** |
| **Respiratory complaint** | **2.64** | **[1.58, 4.39]** |
| **Abnormal AVPU** | **3.10** | **[1.85, 5.19]** |
| **Underweight** | **1.73** | **[1.04, 2.88]** |
